# Pill in the blister pack: a rare cause of dysphagia in an elderly adult

**DOI:** 10.11604/pamj.2015.22.176.8031

**Published:** 2015-10-22

**Authors:** Syed Mudassir Laeeq, Ayesha Aslam Rai, Abbas Ali Tasneem, Nasir Hassan Luck, Zain Majid

**Affiliations:** 1 Department of Hepatogastroenterology, Sindh Institute of Urology & Transplantation, Karachi Pakistan

**Keywords:** Foreign body, pill induced esophageal ulcer, dysphagia

## Abstract

Foreign body impaction in the esophagus amongst adults is not a common cause of dysphagia. Fish bone, food bolus, dentures may cause symptoms of dysphagia, odynophagia, chest pain or respiratory distress. It needs prompt evaluation along with removal of the substance either surgically or endoscopically to avoid the development of life threatening complications. Here we are reporting a case of an elderly male, who presented to us with a history of absolute dysphagia for one week, as a consequence of ingestion of a pill in blister pack.

## Introduction

In adults, true foreign body ingestion (i.e. non-food objects) usually occurs in those having psychiatric disorders, alcohol intoxication, neglected elderly patients and in imprisoned individuals looking for secondary gain via release to a medical facility [1]. Foreign body in the oesophagus is considered to be a grave medical condition, due to possible complications like oesophageal perforation, mediastinitis, fistulization and airway obstruction, carrying a high morbidity and mortality [2].Treatment depends upon the type of material ingested along with the presenting symptoms, therefore early diagnosis and prompt removal via either endoscopic or surgical measure is necessary.

## Patient and observation

**Figure 1:**
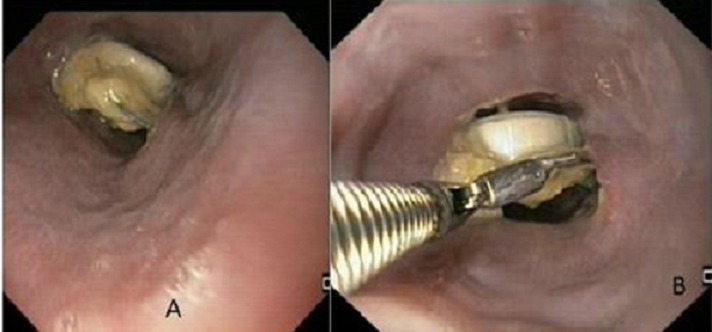
(A) presence of impacted tablets with intact foil; (B) impacted tablet being extracted with the help of biopsy forceps during upper GI endoscopy

A 58 year old male, known smoker, having a ten year history of regular intake of analgesics for arthritis and headache, visited our department via the out patient's department with pain along with difficulty during swallowing since one week. This was more marked for solids than liquids and there was no history of weight loss. He had no recollection of any substance other than the usual meal intake prior to the onset of symptoms. Examination of this patient was unremarkable apart from pallor and epigastric tenderness. Upper gastrointestinal endoscopy was planned and it revealed a tablet in its blister pack at the mid esophagus level (Figure 1 (A)). Two corners of foil wrapper were tucked into the esophageal mucosa, after pulling it with a biopsy forceps, the foreign body was dislodged and later removed. It was identified as tablet Mefenamic acid consistent with his history of analgesics intake. Relook endoscopy was later done, which revealed ulcerated area in lateral wall of mid esophagus (where the sharp ends of foil wrapper were stuck) Figure 1 (B), the lower esophagus and stomach were examined to exclude the presence of other tablets or for possible ulceration. There was no evidence of esophageal perforation and dysphagia and his chest pain got relieved. Patient was kept under observation for 48 hours and he had an uneventful course and was later discharged on high dose proton pump inhibitor (PPI) and sucralfate. Informed consent was obtained from the patient.

## Discussion

Majority of the foreign bodies pass through the gastrointestinal tract without causing any incident and no medical or surgical treatment is required. Endoscopic treatment is necessary in almost 20% of the cases [3]. Swallowing of foreign bodies occurs more frequently in those adult belonging to high risk groups such as prisoners, alcoholics, edentulous adults and psychiatric patients [1]. Our patient did not belong to any of these high-risk categories. According to the available data, frequencies of swallowed foreign bodies vary widely. The foreign bodies most commonly swallowed by adults are fish bones (9-45%), bones (8-40%), dentures (4-18%) [4]. Our patient had swallowed a tablet which was wrapped in its blister pack (foil packing) likely because of dementia. Clinically, foreign body ingestion may cause dysphagia, odynophagia, diffuse chest pain, sensation of chest pressure, laryngeal irritation. Respiratory signs, such as violent coughing, gagging or incomplete airways obstruction may also be present in cases of foreign body aspiration [5]. Our patient presented with a rare cause of absolute dysphagia and chest pain, attributable to ingested pill in blister pack, which was successfully removed endoscopically with the help of biopsy forceps resulting in resolution of dysphagia. Complications attributed to foreign body ingestions are perforation, cervical abscess and mediastinitis [6–8], fortunately because of early intervention and prompt removal of pill via endoscopy had saved our patient from these complications.

## Conclusion

Foreign body ingestion in adults is a reversible cause absolute dysphagia which needs prompt evaluation and removal of substance that may avert possible complications.
